# The making of insulin in health and disease

**DOI:** 10.1007/s00125-020-05192-7

**Published:** 2020-09-07

**Authors:** Jovana Vasiljević, Juha M. Torkko, Klaus-Peter Knoch, Michele Solimena

**Affiliations:** 1 Molecular Diabetology, University Hospital and Faculty of Medicine Carl Gustav Carus, TU Dresden, Dresden, Germany; 2 German Center for Diabetes Research (DZD e.V.), Neuherberg, Germany; 3 Paul Langerhans Institute Dresden (PLID), Helmholtz Center Munich, University Hospital and Faculty of Medicine Carl Gustav Carus, TU Dresden, Tatzberg 47/49, 01307 Dresden, Germany; 4 Max Planck Institute of Molecular Cell Biology and Genetics (MPI-CBG), Dresden, Germany

**Keywords:** Beta cell, Insulin biosynthesis, Insulin maturation, Post-transcriptional regulation, Proinsulin conversion, Review, Type 1 diabetes, Type 2 diabetes

## Abstract

The discovery of insulin in 1921 has been one of greatest scientific achievements of the 20th century. Since then, the availability of insulin has shifted the focus of diabetes treatment from trying to keep patients alive to saving and improving the life of millions. Throughout this time, basic and clinical research has advanced our understanding of insulin synthesis and action, both in healthy and pathological conditions. Yet, multiple aspects of insulin production remain unknown. In this review, we focus on the most recent findings on insulin synthesis, highlighting their relevance in diabetes.

**Electronic supplementary material:**

The online version of this article (10.1007/s00125-020-05192-7) contains a slideset of the figures for download, which is available to authorised users.

**Figure:**
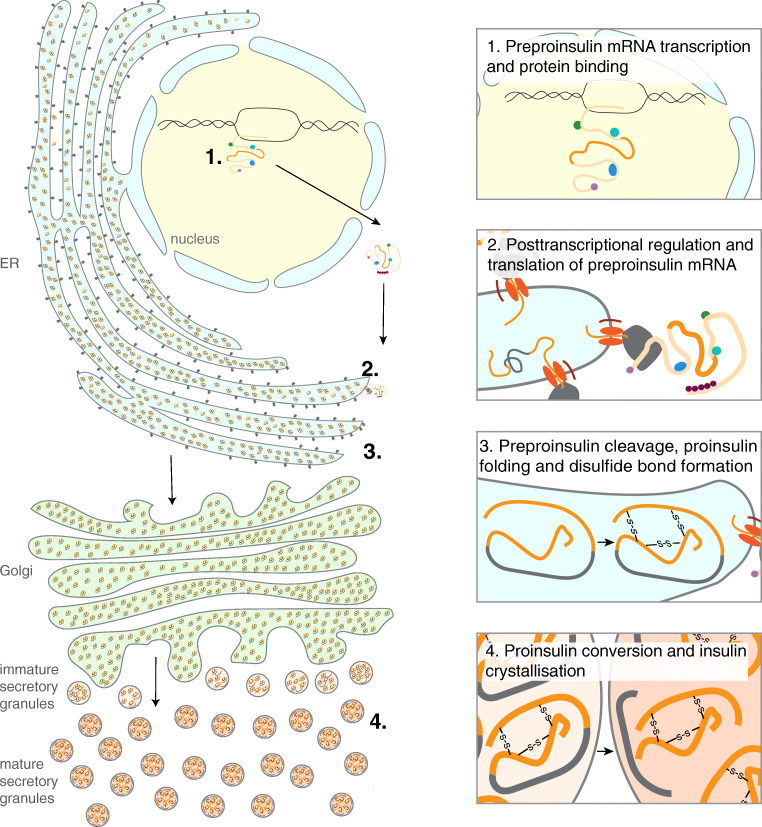
Graphical abstract

## Introduction

Discovered first by Frederik Banting and Charles Best in 1921, insulin is a 51-amino-acid long peptide hormone, which is key for control of glucose homeostasis, metabolism and cell growth [1]. Insulin is thought to be only produced and secreted by the beta cells of the pancreatic islets, although controversial findings have suggested that minute amounts may also be expressed in a subset of neurons in the central nervous system [2]. Within 1–10 min following a meal, hyperglycaemia prompts beta cells to secrete a small fraction (<5%) of their insulin content. Systemic circulation distributes insulin to its main target cells, namely hepatocytes, promoting their glucose storage via glycogen synthesis, and skeletal muscle cells and adipocytes, to stimulate their glucose uptake. Thereby, through these concerted actions, blood glucose is lowered to fasting levels [3].

**Fig. 1:**
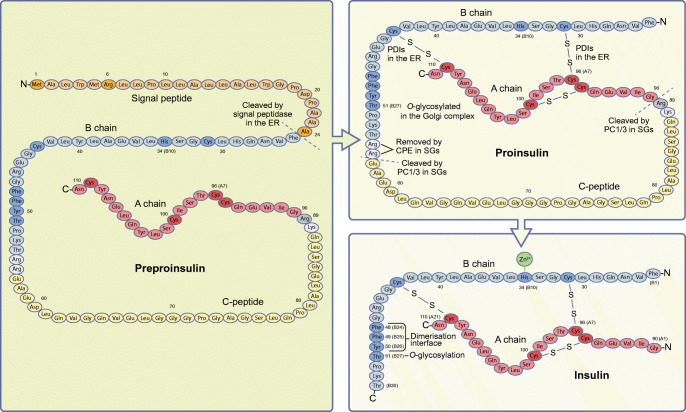
The insulin peptide. Insulin is synthesised as a 110 amino-acid-long preproinsulin including a signal peptide (orange), a B chain (blue), a connecting peptide (C-peptide, yellow) and an A chain (red). The signal peptide targets the preproinsulin to the ER, where it is cleaved by the signal peptidase and converted into proinsulin. In the ER, three disulfide bonds are formed between cysteine residues with the help of PDIs. Proinsulin is trafficked from the ER, through the Golgi and the *trans*-Golgi network to secretory granules (SGs), where PC1/3 and CPE process the dibasic residues (grey) to form mature insulin. Zn^2+^ non-covalently binds to the HisB10 to form the insulin hexamer. Amino acids mentioned in the text are shown in a darker colour. This figure is available as part of a downloadable slideset

**Fig. 2:**
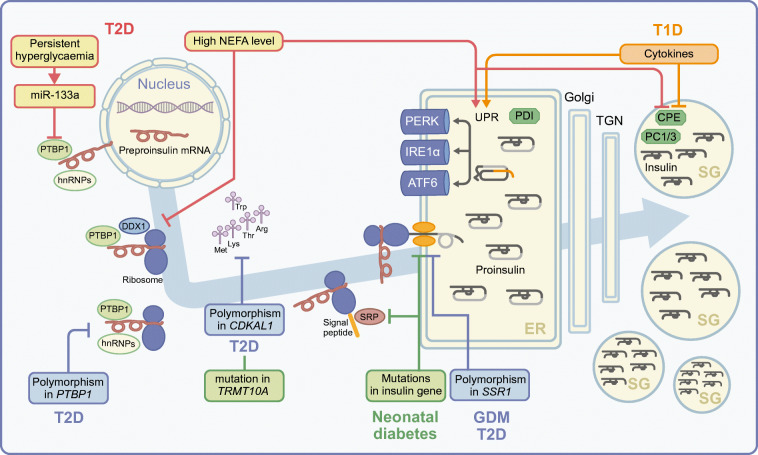
Schematic of insulin production and secretion, from mRNA to the mature hormone. The preproinsulin mRNA is stabilised by its binding to various hnRNPs in the cytosol. Preproinsulin translation and its translocation to the ER starts after the formation and activation of the ribosomal complex. Following proinsulin folding in the ER and the removal of the C-peptide, mature insulin is formed in secretory granules (SGs). Environmental changes, such as metabolic stress or inflammation, that can hamper this highly regulated process are shown in red for type 2 diabetes (T2D) and in orange for type 1 diabetes (T1D); genetic changes resulting in different types of diabetes are labelled in blue (T2D and gestational diabetes mellitus [GDM]) and in green (neonatal diabetes). ATF6, activating transcription factor 6; miR, microRNA; SRP, signal recognition particle; TGN, *trans*-Golgi network. This figure is available as part of a downloadable slideset

Insulin biosynthesis begins with the translation of mRNA into preproinsulin, a polypeptide of 110 amino acids with an N-terminal signal peptide, followed by the B chain, the connecting peptide (C-peptide) and the C-terminal A chain (Fig. 1). Upon translocation into the endoplasmic reticulum (ER), the signal peptide is removed, thereby converting preproinsulin into proinsulin, and disulfide bridges form between the B and A chains. Following its exit from the ER, proinsulin moves through the Golgi complex to the *trans*-Golgi network (TGN) to be sorted into membrane-enclosed organelles termed secretory granules [4] (Fig. 2). Cleavage of the C-peptide in this compartment converts proinsulin into mature insulin, which consists of the B and A chains only (Fig. 1). Mature insulin is stored within secretory granules until they fuse with the plasma membrane to release insulin, or are degraded intracellularly through autophagy or direct delivery to lysosomes, otherwise defined as crinophagy [5–7].

As newly-synthesised insulin is preferentially secreted [8–10], hyperglycaemia simultaneously enhances de novo insulin biosynthesis in order for beta cells to replenish their insulin granule stores and, thus, retain their secretory competence. Given the astonishing assembly rate of >3 × 10^3^ new insulin molecules per second per beta cell [11], each step for insulin production must have been optimised during evolution.

## Glucose regulates insulin mRNA transcription and translation

Glucose is the key factor controlling insulin mRNA expression. Indeed, in glucose-stimulated beta cells, insulin levels are increased 20-fold [12, 13]. This effect is mediated both by transcriptional and post-transcriptional mechanisms. Glucose stimulates the activity of insulin transcription factors pancreatic and duodenal homeobox 1 (PDX-1), neurogenic differentiation 1 (NEUROD1)–E47 and MafA at multiple levels, including changes in their expression levels, subcellular localisation, DNA-binding activity, transactivation capability and interactions with other proteins. For more information on this topic, we refer readers to another review within this special issue [14]. However, for the specific purpose of this article, it is critical to emphasise that the levels of preproinsulin (i.e. unspliced) pre-mRNA and mature (i.e. spliced) preproinsulin mRNA only increase 1 h and several hours after glucose stimulation, respectively [15]. On the other hand, insulin protein levels are already increased 30 min after exposure of rodent and human islets to hyperglycaemia [13, 16, 17]. Taken together, these data indicate that post-transcriptional mechanisms account mostly, if not entirely, for the sudden increase in insulin biosynthesis elicited by glucose stimulation.

Preproinsulin mRNA is the most abundant transcript in beta cells, accounting for ∼30% of their total mRNA content [18]. Resting beta cells store translationally repressed pre-existing copies of preproinsulin mRNA in the cytosol. Hyperglycaemia stimulates the conversion of preproinsulin mRNA into a translationally active form, by altering the combinatorial binding of RNA-binding proteins (RBPs) to *cis-*regulatory elements in its untranslated regions (UTRs). In this way, beta cells can bypass the time-consuming transcriptional step and quickly activate insulin biosynthesis to replenish their stores [19]. In particular, as briefly summarised below, RBPs modulate preproinsulin mRNA stability [16, 18], translation initiation rates [17, 20, 21], including cap-independent translation [22, 23], and transfer to the ER [24].

## Regulation of preproinsulin mRNA stability in health and diabetes

Preproinsulin mRNA has a long half-life, which is mainly regulated by a conserved polypyrimidine tract and a UUGAA-motif in its 3′-UTR [13, 16]. Glucose stimulation increases preproinsulin mRNA stability two- to threefold, as compared with non-stimulated beta cells. The best known RBP that regulates preproinsulin mRNA stability is polypyrimidine tract-binding protein 1 (PTBP1, also known as heterogeneous nuclear ribonucleoprotein [hnRNP] I) [25, 26]. PTBP1 binds to the 3′-UTR of preproinsulin mRNA and prevents its destabilisation by opposing T cell-restricted intracellular antigen 1-related (TIAR) protein [27]. Although it is unclear how, it is known that hyperglycaemia promotes the nucleocytoplasmic translocation and recruitment of PTBP1 to preproinsulin mRNA in the cytosol [28]. Preproinsulin mRNA stability is also enhanced by glucagon-like peptide 1 (GLP-1), which is released from nutrient-stimulated L cells in the gut. Exposure of beta cells to GLP-1 induces the protein kinase A (PKA)-mediated phosphorylation of the nuclear import signal within PTBP1 and, thus, its nucleocytoplasmic translocation [29], conceivably to prime beta cells for the increased insulin demand following a meal.

Other preproinsulin RBPs, at least in insulinoma cells, include hnRNP K [25, 27], hnRNP C, hnRNP E [25], hnRNP L, hnRNP U, HuD [30], and the poly(rC)-binding proteins (PCBP) 1, 2 and 3 [27]. Their involvement in preproinsulin mRNA stability remains unknown and, for some, such as hnRNP K, there are conflicting findings [30]. Yet, several of them are among the most rapidly regulated proteins in INS-1 cells exposed to hyperglycaemia or 3-isobutyl-1-methylxanthine (IBMX) [31], which, like GLP-1, enhances cAMP levels. Hence, evidence increasingly points to RBPs as being critical for rapid post-transcriptional regulation of preproinsulin mRNA.

Since the levels of preproinsulin mRNA in the islets of individuals with normoglycaemia or type 2 diabetes do not significantly differ, its stability is unlikely to be affected in type 2 diabetes [32, 33]. On the other hand, in mouse islets and insulinoma (MIN6) cells exposed to proinflammatory cytokines, no-go and nonsense-mediated RNA decay pathways are upregulated, lowering the levels of preproinsulin mRNA [34]. It is, therefore, possible that inflammation in pancreatic islets in type 1 diabetes alters preproinsulin mRNA stability, while its alternative splicing seems unaffected [35].

## Regulation of preproinsulin mRNA translation in health and diabetes

While a blood glucose concentration of >4–5 mmol/l enhances insulin secretion, glucose concentrations as low as 2–4 mmol/l already support the biosynthesis of insulin to ensure maintenance of its stores [36, 37]. In mammalian cells, translation starts with the binding of various initiation factors to the 5′-UTR of mature mRNAs. This binding occurs in a prescribed order and promotes the recruitment of the small and large ribosomal subunits. Beta cells store preproinsulin mRNA in assembled polysomes. Upon glucose stimulation these polysomes are transported to the ER and preproinsulin mRNA translation starts immediately [24]. Besides common regulators of translation, such as those related to the mammalian target of rapamycin (mTOR) pathway, several other specific factors regulate insulin translation in response to nutrients. Among them is the ATP-dependent RNA helicase DEAD-box helicase 1 (DDX1), which binds to eukaryotic initiation factor (eIF)3a and eIF4b, and to preproinsulin mRNA [38]. These findings could be relevant for the pathogenesis of type 2 diabetes, which is commonly associated with hyperlipidaemia; saturated NEFAs, like palmitate, acutely enhance the secretion of insulin but, unlike its other secretagogues (glucose and GLP-1), they do not concomitantly increase its production [39]. In mice, in particular, palmitate-induced phosphorylation of DDX1 displaces it from the preproinsulin mRNA and suppresses insulin biosynthesis, hence providing a direct link between hyperlipidaemia and insulin deficiency [38]. Moreover, depletion of Ca^2+^ levels in the ER upon exposure to palmitate can impair proinsulin folding and cause ER stress, hence further downscaling insulin biosynthesis [40].

Transfer RNAs (tRNAs) deliver amino acids to translating ribosomes, and their post-transcriptional methylation enhances the fidelity and, thus, efficiency of translation. Polymorphisms or mutations in tRNA methyltransferases, such as in *CDKAL1* and *TRMT10A* [41–43], are associated with glucose intolerance due to impaired insulin synthesis and, in the case of *TRMT10A*, cause a monogenetic form of young-onset diabetes associated with microencephaly and intellectual disability. Interestingly, the use of an alternative start codon in human preproinsulin *mRNA* can lead to the translation of a nonconventional insulin product and the generation of neoantigenic peptides thereof, which are targets of T cell-mediated autoimmunity in type 1 diabetes [44].

All eukaryotic mRNAs are capped at their 5′-UTR and translated in a cap-dependent fashion. However, cap-independent translation can occur if initiation factors are recruited closer to the first AUG codon through an internal ribosome entry site (IRES). Bypassing many of the controls for cap-dependent translation, cap-independent translation allows for protein synthesis in conditions in which the former is compromised, for example, upon irradiation, hypoxia, apoptosis or amino-acid starvation. Notably, mRNAs for insulin and other secretory granule cargoes can be translated in a cap-independent manner, thus enabling their continuous production, even in stress conditions [23] or upon inhibition of the mTOR pathway [45]. Key to this process is PTBP1, the binding of which to the preproinsulin mRNA 5′-UTR is increased upon transient hyperglycaemia. However, exposure of human islets to prolonged hyperglycaemia suppresses PTBP1 expression and insulin biosynthesis, possibly due to the concomitant upregulation of microRNA (miR)-133a, which binds to the 3′-UTR of PTBP1 mRNA [46]. Increased nuclear retention of PTBP1 in the islets of individuals with type 2 diabetes may also contribute to impaired glucose-stimulated insulin biosynthesis [47]. Furthermore, common polymorphisms within *PTBP1* influence glucose-stimulated insulin secretion [48], albeit, in general, *PTBP1* mRNA levels in the islets of individuals with impaired glucose tolerance and type 2 diabetes are unaffected as compared with individuals with normoglycaemia [33].

Although most mutations in insulin’s amino acid sequence impair its folding in the ER [49–51] (see below), there are also single-point mutations (such as the three shown in dark orange in Fig. 1) or extensive exon deletions that affect its efficient translation (Fig. 1). In all these instances, translation initiation of preproinsulin is immediately arrested, leading to permanent neonatal diabetes. For instance, mutations in the start codon of preproinsulin cause immediate arrest of translation initiation. Downstream mutations in the signal peptide of preproinsulin, such as R6C replacement [52], also impair ER translocation and target preproinsulin for proteasomal degradation. Furthermore, mutations affecting signal-peptide cleavage, such as the A24D replacement, also cause permanent neonatal diabetes by blocking ER exit of the protein and leading to ER stress [50, 51]. Finally, polymorphisms in the gene encoding translocon-associated protein subunit α (*SSR1*) may alter preproinsulin translocation and, thus, predispose carriers to type 2 diabetes and gestational diabetes [53, 54].

## Regulation of proinsulin folding in health and diabetes

Proinsulin folding in the ER involves the establishment of three disulfide bonds, two interchain between the B and A chains and one intrachain within the A chain (Fig. 1). Altered cysteine pairing, such as upon mutations of C96 in the A chain, causes misfolding, accumulation and toxic aggregation of proinsulin in the ER leading to permanent neonatal diabetes or mature-onset diabetes of the young (reviewed previously [50]). Recent studies in a human-derived induced pluripotent stem cell (iPSC) model of neonatal diabetes with a C96R mutation, and in the Akita mouse model of diabetes, in which one *Ins2* allele carries a C96Y replacement, suggest that proinsulin misfolding reduces beta cell proliferation and mass due to downregulation of mTOR signalling during pancreas development [55–57], rather than beta cell apoptosis, as it is commonly assumed.

In vitro studies suggest that among the 15–20 protein disulfide isomerases (PDIs) found in the human genome, PDIA1 primarily facilitates the oxidative folding of proinsulin disulfide bonds in conjunction with the oxidoreductases ER oxidoreductin 1α/β (ERO1α/β) [51, 58, 59]. In turn, ERO1α/β regenerate PDIs for subsequent rounds of disulfide bond generation. Increasing evidence suggests that modest amounts of proinsulin disulfide mispairing occurs even in healthy beta cells and that accumulation of misfolded proinsulin intermediates occurs early in type 2 diabetes [60, 61]. Thus, maintenance of a proper redox status, which relies on the continuous supply of reducing equivalents by the cytosolic thioredoxin system, is critical for proper beta cell function. Interestingly, the thioredoxin interacting protein (TXNIP), which inhibits the antioxidative action of thioredoxin and is elevated upon ER stress and insulin misfolding [62, 63], was also found to be elevated in type 2 diabetes islets [64].

Perturbance of proinsulin folding is countered by the unfolded protein response (UPR). Among the three UPR sensors, namely inositol-requiring enzyme 1α (IRE1α), protein kinase RNA-like ER kinase (PERK) and activating transcription factor 6 (ATF6), IRE1α and PERK have been more extensively implicated in the regulation of proinsulin folding. IRE1α signalling is constitutively active under physiological conditions for control of oxidative proinsulin folding [58], whereas the PERK pathway is induced upon ER stress [65, 66]. Mutations in *PERK* (also known as *EIF2AK3*) cause the Wolcott–Rallison syndrome, an autosomal recessive permanent neonatal diabetes [65]. On the other hand, inactivating mutations of the heat shock protein p58^IPK^, resulting in increased PERK activity, can also cause diabetes [51, 65]. In type 1 diabetes, cytokine-induced ER stress may account for impaired folding of insulin and altered HLA presentation of antigenic peptides thereof, hence contributing to beta cell dysfunction and T cell-mediated destruction [67–69].

## Regulation of proinsulin conversion into mature insulin in health and diabetes

The conversion of proinsulin into mature insulin occurs in two consecutive steps; first, the C-peptide junctions at the B and A chains are cleaved at basic residues R55–R56 and K88–R89 (Fig. 1) [70, 71]. In human beta cells, proprotein convertase 1/3 (PC1/3) is mainly responsible for C-peptide release, while in rodent beta cells, its paralogue proprotein convertase 2 (PC2) also participates in this process [72]. Next, the exopeptidase carboxypeptidase E/H (CPE) removes the dibasic residues R55–R56 at the C-terminal end of the B chain (Fig. 1) [70, 71]. Although impaired proinsulin conversion with elevated proinsulin secretion is a hallmark of type 2 [73] and type 1 diabetes [74, 75], genetic variants affecting the junction between the C-peptide and the A chain, such as replacement of R89 [50] (Fig. 1), or the proteolytic activities of either PC1/3 [76] or CPE [77] have only been identified in a few individuals with type 2 diabetes or altered glucose metabolism. While islets of donors with type 2 diabetes do not display reduced expression of *PC1/3* (also known as *PCSK1*) and/or *CPE* mRNA [33, 78], one study found that palmitate treatment of islets isolated from non-diabetic deceased organ donors reduced CPE protein levels [79]. This intriguing observation remains to be validated in studies of CPE expression in islets of individuals with type 2 diabetes in situ. On the other hand, proteomic analysis indicated that PC1/3 and CPE are reduced in islets from donors with type 1 diabetes that were obtained by laser capture microdissection (LCM) [80, 81]. Besides reduced expression of PC1/3 and CPE, other mechanisms could account for the inefficient conversion of proinsulin into mature insulin. The activation of PC1/3 and CPE, which also travel through the secretory pathway together with proinsulin, is induced by the lowering of luminal pH to <6.0 and the rise of Ca^2+^ concentration in immature insulin secretory granules. Therefore, reduced proinsulin-to-insulin conversion may also reflect changes in luminal acidification, for instance, due to premature ageing of secretory granules, since the pH of older granules is ≥6.2 (M. Neukam and M. Solimena, unpublished results). Beta cell degranulation, due to excessive insulin demand, may also force the immediate release of immature secretory granules without providing enough time for efficient proinsulin-to-insulin conversion. This scenario, however, remains to be proven.

In most mammalian beta cells, including humans, mature insulin is stored in secretory granules as a hexamer of three dimers, each of which coordinates the binding of a Zn^2+^ molecule to H34 (B10) in the B chain (Fig. 1). Albeit, a notable exception to this is guinea pig insulin, which does not bind to Zn^2+^. Import of Zn^2+^ into secretory granules is mediated by the zinc transporter 8 (ZnT8; encoded by the *SLC30A8*), a known autoantigen of type 1 diabetes and a risk gene for type 2 diabetes. Depletion of Zn^2+^ impairs both insulin crystallisation, thereby altering the characteristic appearance of the granule-dense core that can be seen using electron microscopy, and insulin secretion in mice [82]. Remarkably, carriers of the ZnT8 variant R325W, which correlates with lower expression of the transporter, convert proinsulin to insulin more efficiently and have a lower risk of developing type 2 diabetes. On the other hand, in these individuals, the import of Zn^2+^ into secretory granules may be compensated by other zinc transporters [83, 84].

The dimerisation interface of the insulin B chain, which contains the aromatic triplet F48, F49 and Y50 (Fig. 1), facilitates the sorting and maturation of proinsulin and the interaction of insulin with its receptor [49, 85–87]. Intriguingly, large-scale analysis of human and mouse islets by targeted mass spectrometry have only very recently revealed that the adjacent T51 *is O-*glycosylated (Fig. 1) [88]. The functional implications of this modification, however, remain to be determined.

## Conclusion and some outstanding questions

As we briefly summarised above, in recent years our understanding of post-transcriptional and translational mechanisms for insulin production and their impairment in diabetes has progressed relentlessly. Still, numerous aspects of these processes remain unclear. For instance, factors involved in the regulation of splicing and nucleocytoplasmic transport of preproinsulin mRNA are unknown. Also unknown is where exactly in the cytosol resting beta cells store untranslated preproinsulin mRNA, while our knowledge about the machinery regulating its stability and degradation is rudimental. In vitro studies and animal models suggest that ER stress and proinsulin traffic are relevant to the pathogenesis of type 1 and type 2 diabetes, but conclusive evidence in humans is still missing. We also lack a clear explanation for the inefficient processing and elevated release of proinsulin in type 2 diabetes. The same post-transcriptional and translational mechanisms likely coordinate the biosynthesis of other insulin granule cargoes and enable the proper assembly of these organelles [3, 26]. As exemplified by the occurrence of diabetes in carriers of mutations affecting insulin production, any deficit along this supply chain can deplete beta cells of new insulin granule stores, thereby hampering their competence for glucose-stimulated secretion. Insulin and other insulin granule cargoes are also major targets of autoimmunity in type 1 diabetes. The inherent reasons for deficient insulin secretion in type 2 diabetes and for autoimmunity against insulin granule components are, however, yet to be discovered. Thus, despite popular belief suggesting that by now we know how cells manufacture insulin [89], almost a century after the discovery of insulin, much remains to be uncovered regarding its production and release for the control of glucose homeostasis. We are nonetheless confident that ingenuity and access to ever new powerful methodologies will enable this knowledge gap to be filled.

## Electronic supplementary material

Slideset of figures (PPTX 647 kb)

## Abbreviations

CPE: Carboxypeptidase E/H
DDX1: DEAD-box helicase 1
eIF: Eukaryotic initiation factor
ER: Endoplasmic reticulum
ERO1α/β: Endoplasmic reticulum oxidoreductin 1α/β
GLP-1: Glucagon-like peptide 1
hnRNP: Heterogeneous nuclear ribonucleoprotein
IRE1α: Inositol-requiring enzyme 1α
mTOR: Mammalian target of rapamycin
PC1/3: Proprotein convertase 1/3
PDI: Protein disulfide isomerase
PERK: Protein kinase RNA-like endoplasmic reticulum kinase
PTBP1: Polypyrimidine tract-binding protein 1
RBP: RNA-binding protein
tRNA: Transfer RNA
UPR: Unfolded protein response
UTR: Untranslated region
