# Supplementary material for: The making of insulin in health and disease
Source: Diabetologia. 2020 Sep 7;63(10):1981–9. doi: 10.1007/s00125-020-05192-7 (PMC7476993; doi:10.1007/s00125-020-05192-7)
Supplement: Supplementary file 1 — (PPTX 647 kb) [file 125_2020_5192_MOESM1_ESM.pptx]

## Slide 1
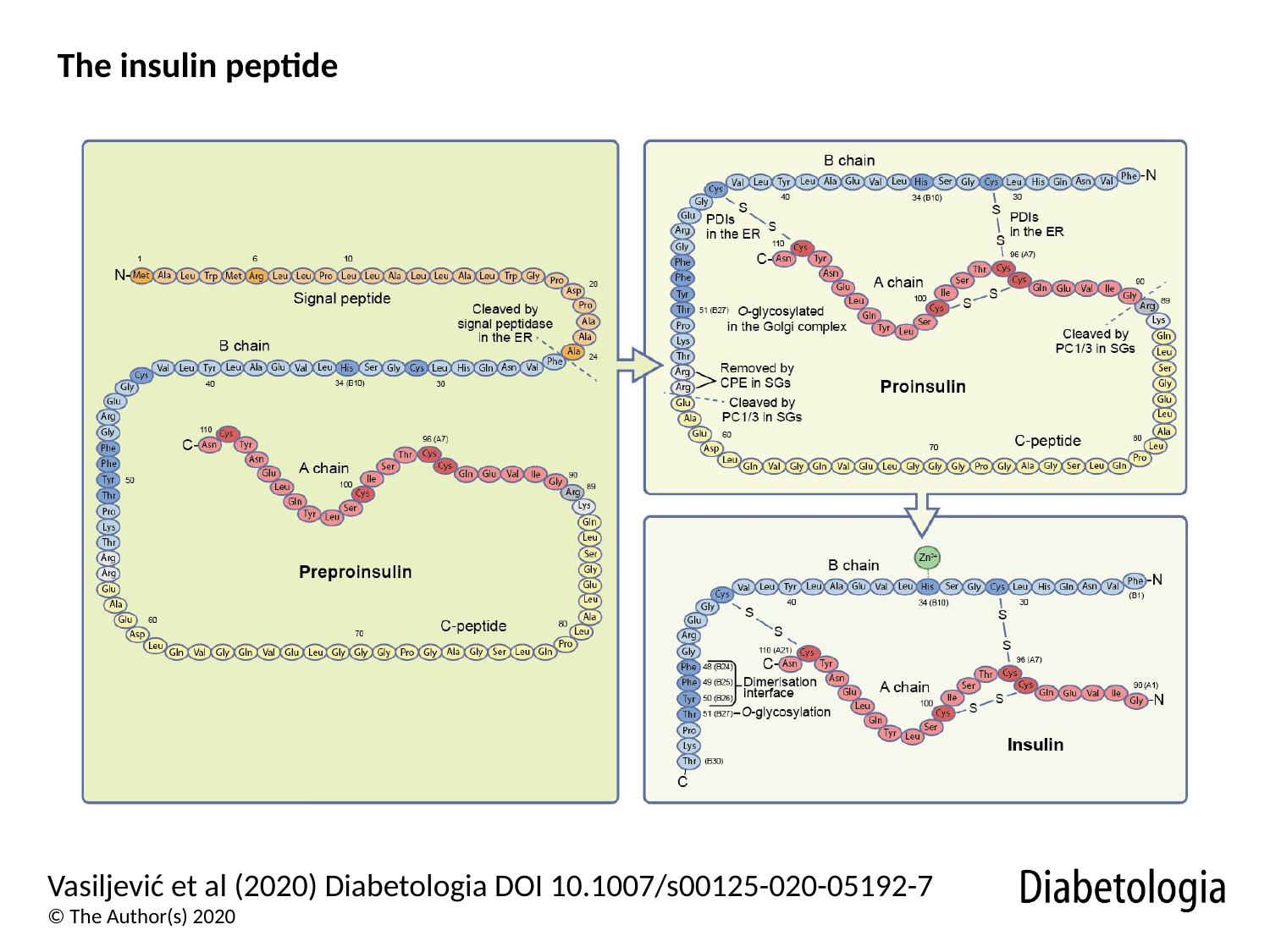

The insulin peptide
Vasiljević et al (2020) Diabetologia DOI 10.1007/s00125-020-05192-7
© The Author(s) 2020

## Slide 2
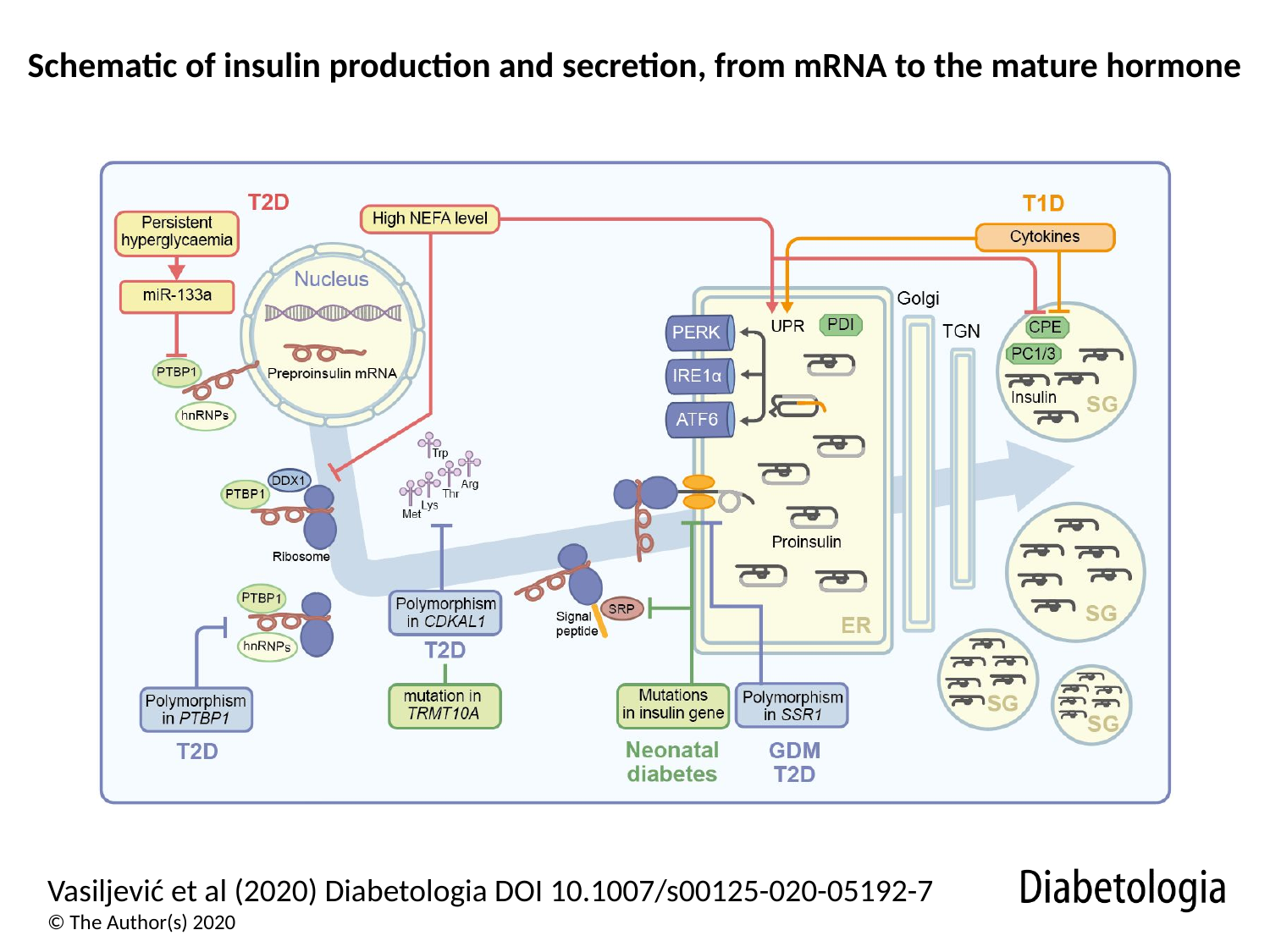

Schematic of insulin production and secretion, from mRNA to the mature hormone
Vasiljević et al (2020) Diabetologia DOI 10.1007/s00125-020-05192-7
© The Author(s) 2020
